# Transforming Retinal Photographs to Entropy Images in Deep Learning to Improve Automated Detection for Diabetic Retinopathy

**DOI:** 10.1155/2018/2159702

**Published:** 2018-09-10

**Authors:** Gen-Min Lin, Mei-Juan Chen, Chia-Hung Yeh, Yu-Yang Lin, Heng-Yu Kuo, Min-Hui Lin, Ming-Chin Chen, Shinfeng D. Lin, Ying Gao, Anran Ran, Carol Y. Cheung

**Affiliations:** ^1^Department of Electrical Engineering, National Dong Hwa University, Hualien, Taiwan; ^2^Department of Medicine, Hualien Armed Forces General Hospital, Hualien, Taiwan; ^3^Department of Medicine, Tri-Service General Hospital, National Defense Medical Center, Taipei, Taiwan; ^4^Department of Electrical Engineering, National Sun Yat-sen University, Kaohsiung, Taiwan; ^5^Department of Electrical Engineering, National Taiwan Normal University, Taipei, Taiwan; ^6^Department of Computer Science and Information Engineering, National Dong Hwa University, Hualien, Taiwan; ^7^Department of Medicine, University of California, San Francisco, CA, USA; ^8^Department of Ophthalmology and Visual Sciences, Chinese University of Hong Kong, Sha Tin, Hong Kong

## Abstract

Entropy images, representing the complexity of original fundus photographs, may strengthen the contrast between diabetic retinopathy (DR) lesions and unaffected areas. The aim of this study is to compare the detection performance for severe DR between original fundus photographs and entropy images by deep learning. A sample of 21,123 interpretable fundus photographs obtained from a publicly available data set was expanded to 33,000 images by rotating and flipping. All photographs were transformed into entropy images using block size 9 and downsized to a standard resolution of 100 × 100 pixels. The stages of DR are classified into 5 grades based on the International Clinical Diabetic Retinopathy Disease Severity Scale: Grade 0 (no DR), Grade 1 (mild nonproliferative DR), Grade 2 (moderate nonproliferative DR), Grade 3 (severe nonproliferative DR), and Grade 4 (proliferative DR). Of these 33,000 photographs, 30,000 images were randomly selected as the training set, and the remaining 3,000 images were used as the testing set. Both the original fundus photographs and the entropy images were used as the inputs of convolutional neural network (CNN), and the results of detecting referable DR (Grades 2–4) as the outputs from the two data sets were compared. The detection accuracy, sensitivity, and specificity of using the original fundus photographs data set were 81.80%, 68.36%, 89.87%, respectively, for the entropy images data set, and the figures significantly increased to 86.10%, 73.24%, and 93.81%, respectively (all *p* values <0.001). The entropy image quantifies the amount of information in the fundus photograph and efficiently accelerates the generating of feature maps in the CNN. The research results draw the conclusion that transformed entropy imaging of fundus photographs can increase the machinery detection accuracy, sensitivity, and specificity of referable DR for the deep learning-based system.

## 1. Introduction

Diabetic retinopathy (DR) is one of the microvascular complications related to diabetes mellitus and a major cause of blindness globally. In the United States, the DR prevalence among diabetic patients is between 20% and 30% [1, 2]. Fundus photography is a direct visual screening tool used to detect DR and has been widely accepted worldwide. However, the detection of DR and assessment of its severity require specialized expertise, and the agreement of interpretation results between examiners varied substantially based on previous studies [3–5]. In addition, many diabetic patients do not have access to effective screening programs and some cannot afford the cost of an ophthalmologist visit [6]. Deep learning is a subset of machine learning, and in modern medicine, using deep learning in fundus photography has emerged as a cost-effective and practical method for automated grading of DR [5, 7, 8].

To implement deep learning in fundus photography for DR grading, a large data set of fundus photography is required, and the amount of data for each grade is preferred to be evenly distributed. However, the retinal images collected from different eye clinics are not standardized (e.g., differences in contrast, brightness, and file size) in all known open web data sets and epidemiology reports [8–10]. Therefore, it is important to preprocess the original images to increase the heterogeneity before putting the images into the training for the automated detection of DR. Several preprocessing methods have been applied to the deep learning of fundus photography [5, 11, 12]. Data augmentation would generate training images utilizing different processing skills or a combination of these skills, such as rotating, shifting, and flipping to the original training images. In addition, contrast and brightness adjustment would increase the heterogeneity and accuracy of the testing, and therefore enhance the automated grading performance. These currently available preprocessing methods can efficiently increase the number of original fundus photographs used for deep learning, without changing the morphology of most predictive features. As the original retinal images are preprocessed or translated to another form of quantitative bioinformative images, they are in essence considered new images by the CNN, so the performance of deep learning will be improved.

Theoretically, severe DR leads to higher heterogeneity than mild or no DR in a fundus photograph. Image entropy, which involves measuring the complexity of an image, may be a good preprocessing method to increase the heterogeneity of fundus photographs [13]. Image entropy can be utilized to describe the total amount of information. There exist differences between no or mild DR and severe DR images from the distributions of localized image entropy [13]. To distinguish the features of no or mild DR and severe DR, we analyzed the complexity of fundus photographs by calculating local image entropy. Images of low entropy have low contrast level, and, on the contrary, high entropy stands for high contrast level between neighboring pixels. DR images contain papilledema and retinal capillary leakage with high entropy values; however, no or mild DR images may have more flat areas with low local entropy. In this study, we hypothesized that, for this deep leaning application, transforming the original fundus photographs to entropy images may improve the performance, including accuracy, sensitivity, and specificity of detecting the presence of the more severe grade of DR.

## 2. Methods

### 2.1. Data Sets

The “Kaggle Diabetic Retinopathy” data set collected large numbers of fundus photographs from diabetic patients for a competition to detect DR by different deep learning algorithms at early 2015. The data set was publicly available and retrieved in 2017 for images training and testing in this study [14]. In summary, there are total 35,126 color fundus photographs, of which the sizes range from 433 × 289 pixels to 5184 × 3456 pixels [14–17]. The photographic quality was defined as poor if the image was subjectively blurred, and objectively not covered both the regions of fovea and optic disc. As a result, we removed 14,003 images of poor quality and kept 21,123 fundus photographs for the following experiments. These training photographs of the Kaggle data set were obtained using various digital fundus cameras in multiple eye centers in California and around the United States. The retina data set is provided by Kaggle's diabetic retinopathy detection competition. Kaggle, a subsidiary company of Google, is known to be the biggest platform of data science community and has held over 200 competitions. In this data set, 661 teams participated in this diabetic retinopathy detection competition. In addition, the retina data set is provided by EyePACS, which is an efficacious store-and-forward clinical communication system that has been tested with clinical trials. To sum up, this data set is a representative data set for diabetic retinopathy detection.

### 2.2. Grading

In the experimental images obtained from the Kaggle data set, the severity of DR was independently graded by well-trained clinicians according to the International Clinical Diabetic Retinopathy scale: no DR (unaffected, Grade 0, *n*=16,500, 78.11%), mild nonproliferative DR (Grade 1, *n*=1,333, 6.31%), moderate nonproliferative DR (Grade 2, *n*=2,000, 9.47%), severe nonproliferative DR (Grade 3, *n*=645, 3.05%), and proliferative DR (Grade 4, *n*=645, 3.05%) [18]. The presence of severe DR of grades 2–4 that requires a referral to a specialist was defined as referable DR (*n*=3,290), accounting for 15.6% of the original images. Referable DR was used for the deep learning in this study.

### 2.3. Preprocessing of Images

To standardize the image condition, several preprocessing steps were carried out for the original fundus photographs before conducting the training for deep learning. First, image pixels with values between 0 and 255 were scaled to have values between 0 and 1. Images were then downsized to a standard resolution of 100 × 100 pixels.

Second, in order to balance the number of images with no DR for effective deep learning on features, the number of images with DR was increased evenly for Grade 1 to Grade 4 (4,375, 4,375, 3,875, and 3,875, respectively) by rotating and flipping their original images, for a total of 16,500 images. We randomly selected 15,000 images without DR (Grade 0), and 15,000 images with DR (*n* = 4,000, 4,000, 3,500, and 3,500, respectively, for Grade 1 to Grade 4) for a total of 30,000 images in the training data set. In addition, the remaining 1,500 images without DR (Grade 0), and the 1,500 images with DR (*n* = 375, 375, 375, and 375, respectively, for Grade 1 to Grade 4) were used as the testing data set.

### 2.4. Preprocessing to Entropy Images

Entropy images were computed from *n* × *n* blocks of the luminance of the original fundus photograph based on the block spatial scale responses. All of the entropy values were computed locally [19] using the software Matlab v.8.2. Spatial entropy is a function of the probability distribution of the local gray values. Equation (1) of the local entropy image is described as follows:

(1) $$\begin{array}{c} E_{\text{local}}=-\underset{i}{\sum }P\left(i\right)\times \log_{2}\;P\left(i\right). \end{array}$$

In the probability density function, *P*(*i*) denotes the relative frequency associated with the *i*-th gray level within a *n* × *n* block. In this study, the entropy values were calculated using *n* = 2, 3, 5, 9, and 11 in the experiments.

The entropy images, as with the original fundus photographs, were also downsized to a standard resolution of 100 × 100 pixels. This method analyzes the local entropy and utilizes the statistical characteristics of the local regions, which helps exhibit the local structural information of the image [19].

### 2.5. Deep Feature Learning

**Figure 1:**
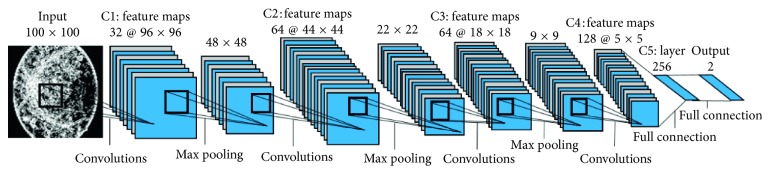
The diagram of all layers in CNN.

Convolutional neural networks (CNNs) [20], which contain architecture consisting of multiple layers for deep learning, have been widely applied in a large number of image recognition tasks; the CNN with different parameters are trained and used for the feature learning of referable DR of both fundus photographs and entropy images, respectively, in this study. As it has been known, the common layers of CNN include convolutional layer, pooling layer, rectified linear unit (ReLU) layer, dropout layer, fully connected layer, and classification layer. Successive convolutional layers transform the input images into serial feature maps through iterative filters and learn to recognize features at differing spatial levels automatically. Every layer of a deep learning system receives the data from the adjacent upper layer and produces a presentation of the observed pattern, then transmits it to the adjacent lower layer.  Figure 1 shows a diagram of all layers in this CNN.

In this study, we used 4 convolutional layers of the same kernel size (5 × 5), and the number of filters in each convolutional block is 32, 64, 64, and 128 in successive layers. Maximal pooling layers are placed in the first 3 convolutional layers to partition the feature maps and collect the maximal value in the subimage. In deep convolutional networks framework, each convolutional layer uses ReLU activation function and adds a dropout layer to prevent overfitting. The CNN was implemented by TensorFlow software, and the number of iterations for the training procedure was 200. The best results of detection accuracy were, respectively, selected among the 200 iterations for original images and entropy images to prevent the potential bias.

### 2.6. Statistical Analysis and Performance Comparison

The performance of CNN was evaluated by the detection accuracy, sensitivity, and specificity of the automated interpretation for referable DR presence; the clinically defined referable DR in the Kaggle data set was used as the benchmark. A comparison of the accuracy was performed by using a chi-square test. In addition, a comparison between the original fundus photographs and the entropy images for the sensitivity and specificity was performed by using a McNemar's test. Furthermore, the area under the receiver-operating characteristic curve (AUC) was also used to evaluate and compare the discrimination of the machinery interpretation for referable DR presence between the original retinal photographs and entropy images. A 2-tailed value of *p* < 0.05 was considered statistically significant. Analyses were performed using SAS statistical software (version 9.4, SAS Institute Inc, Cary, NC).

The indexes are defined in Equations (2)–(4). TP, TN, FP, and FN represent true positive, true negative, false positive, and false negative, respectively.

(2) $$\begin{array}{c} \text{accuracy}=\frac{\text{TP}+\text{TN}}{\text{TP}+\text{TN}+\text{FN}+\text{FP}}, \end{array}$$

(3) $$\begin{array}{c} \text{sensitivity}=\frac{\text{TP}}{\text{TP}+\text{FN}}, \end{array}$$

(4) $$\begin{array}{c} \text{specificity}=\frac{\text{TN}}{\text{TN}+\text{FP}}. \end{array}$$

## 3. Results

**Figure 2:**
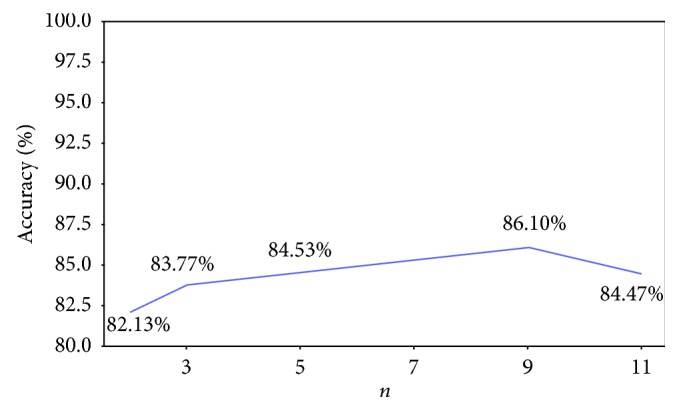
The distribution of accuracy vs. various block sizes (*n*) for the detection of referable DR of the entropy images.

Figure 2 compares the accuracy of entropy images with various block sizes (*n*) for the detection of referable DR. The results of *n*=9 reach the maximal accuracy of 86.10% among the various block sizes. Accordingly, *n*=9 was selected to calculate the entropy images in our experiments.

**Figure 3:**
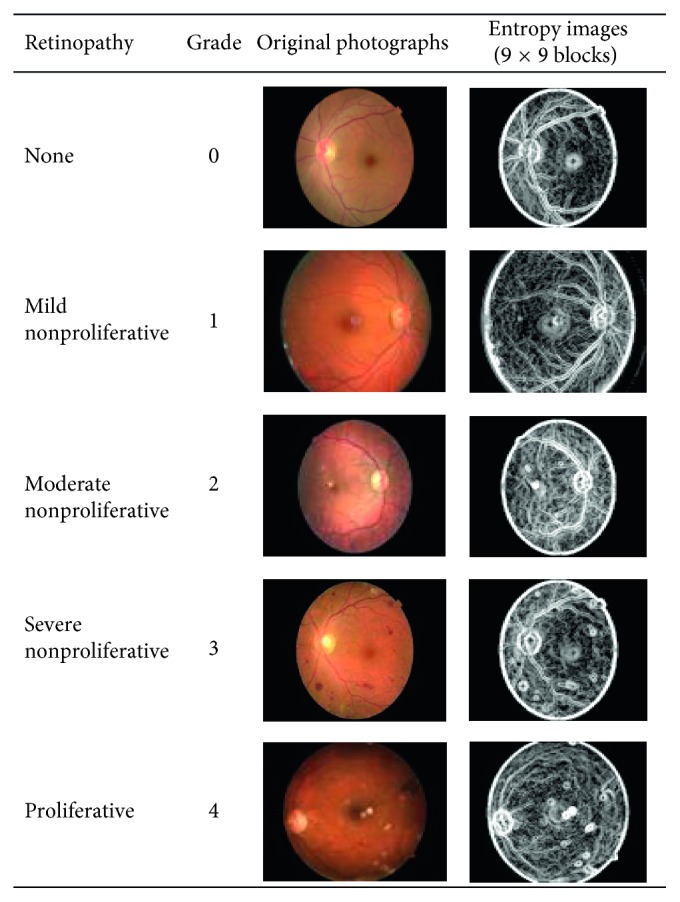
Original fundus photographs and entropy images of DR of any grade (0–4).

Figure 3 illustrates some examples of the original fundus photographs of Grade 0 to Grade 4 DR and their entropy images when block size *n* is 9.  Table 1 reveals that the detection accuracy, sensitivity, and specificity for referable DR in the original photographs were 81.80%, 68.36%, and 89.87%, respectively, and they increased to 86.10%, 73.24%, and 93.81%, respectively, with the entropy images data set when *n* is 9.

**Figure 4:**
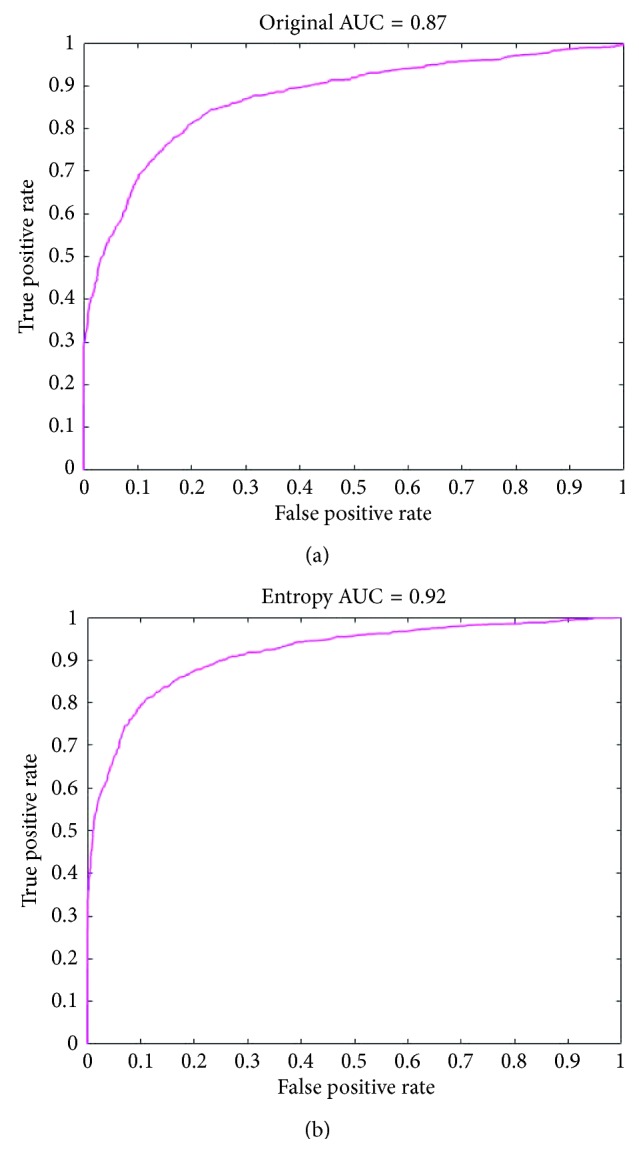
The AUC for the discrimination of automated interpretation for referable DR in (a) original photographs and (b) entropy images.

The AUC of the deep learning for the detection of referable DR in the original fundus photographs and the entropy images data sets was 0.87 and 0.92 (*n*=9), respectively (*p* < 0.001), as shown in Figures 4(a) and 4(b).

## 4. Discussion

Our study demonstrates that, compared with the original fundus photographs, the entropy images can improve the deep learning performance and correctly detect the clinician-defined referable DR (accuracy 81.80% and 86.10%). Our result is also consistent with previous studies [5, 8, 11] that deep feature learning may be an effective method to provide an estimation of referable DR for the original photographs and entropy images (sensitivity: 68.36% and 73.24% and specificity: 89.87% and 93.81%, respectively).

Several preprocessing methods for fundus photographs have been proposed to improve the performance of deep learning in the detection of DR. For instance, extraction of blood vessels from fundus photographs at baseline may improve the accuracy of detecting referable retinopathy after deep learning [21]. These preprocessing methods preserve vital characteristics of retinopathy lesions on the background of normal retinal anatomy for CNN training. On the contrary, the entropy images represent an index of the complexity of the original fundus photographs, probably resulting from the increased number of DR lesions in contrast to the neighboring retinal vessels. In addition, we find that the best result of detection performance by CNN is achieved when block size = 9 for the generation of entropy images. The empirical selection reflects the suitable block size for the enhancement of local image structure for fundus photographs to distinguish the features of DR image. Our study confirms that signs of higher grade retinopathy may have higher complexity in fundus photograph. Although the detection performance of CNN learning for the detection of referable DR is only modestly improved when using entropy images (the net improvement rate in accuracy is 4.3%), the evidence is sufficient to demonstrate that transforming fundus photographs to entropy images may be useful in deep learning for referable DR.

Previous studies using the Kaggle Diabetic Retinopathy and other publicly available data sets have shown that there are high AUC levels and sensitivity: 90–100% [22–25] and a wide range of specificity: 50–97.6% in the CNN learning for any or referable DR in the original fundus photographs [16, 21–24]. Similarly, we have a high AUC (0.87) for referable DR in the original fundus photographs. However, compared with previous study findings [16, 21–25], our results show relatively low sensitivity (68.36%) and high specificity (89.87%) in the original photographs. Since we merely selected the Kaggle training data set as our experimental samples, the difference in the sensitivity and specificity between our study and those of others might be due to a relatively lower prevalence of referable DR in the Kaggle training data set than in other data sets, or the unequal distribution of each DR grade in the process of augmenting DR images. The strength of our study is we use a well-known and large-sized publicly available fundus photography data set for deep learning, and we can reproduce the results and compare our findings with other studies.

In conclusion, the entropy image quantifies the amount of information in the retinal photograph and can efficiently accelerate the generation of feature map in CNN. Preprocessed entropy imaging of retinal photographs may increase the machinery detection accuracy, sensitivity, and specificity of referable DR for a deep learning-based system.

## Figures and Tables

**Table 1:** The performance between the original photographs and entropy images.

	Original photographs (%)	Entropy images (%)	*p* value
Accuracy	81.80	86.10	<0.001
Sensitivity	68.36	73.24	<0.001
Specificity	89.87	93.81	<0.001

## Data Availability

The data used to support the findings of this study are available from the corresponding author upon request.
